# Biochemical characterization of a phospholipase A_2_ homologue from the venom of the social wasp *Polybia occidentalis*

**DOI:** 10.1186/s40409-018-0143-1

**Published:** 2018-02-15

**Authors:** Rafaela Diniz-Sousa, Anderson M. Kayano, Cleópatra A. Caldeira, Rodrigo Simões-Silva, Marta C. Monteiro, Leandro S. Moreira-Dill, Fernando P. Grabner, Leonardo A. Calderon, Juliana P. Zuliani, Rodrigo G. Stábeli, Andreimar M. Soares

**Affiliations:** 1 Center for the Study of Biomolecules Applicable to Health (CEBio), Oswaldo Cruz Foundation – Rondônia (Fiocruz – Rondônia), Porto Velho, RO Brazil; 2 Department of Medicine, Federal University of Rondônia (UNIR), Porto Velho, RO Brazil; 3 Postgraduate Program in Experimental Biology (PGBIOEXP), Federal University of Rondônia (UNIR), Porto Velho, RO Brazil; 4 São Lucas University Center (UniSL), Porto Velho, RO Brazil; 5 Postgraduate Program in Biodiversity and Biotechnology, Bionorte Network, Federal University of Rondônia (UNIR), Porto Velho, RO Brazil; 6 School of Pharmacy, Federal University of Pará (UFPA), Belém, PA Brazil; 7 Department of Medicine, UFSCar, São Carlos, Center of Translational Medicine, Fiocruz – SP, and School of Medicine of Ribeirão Preto, University of São Paulo (USP), São Paulo, Brazil

**Keywords:** Wasp, *Polybia occidentalis*, PocTX, PLA_2_ homologue

## Abstract

**Background:**

Wasp venoms constitute a molecular reservoir of new pharmacological substances such as peptides and proteins, biological property holders, many of which are yet to be identified. Exploring these sources may lead to the discovery of molecules hitherto unknown. This study describes, for the first time in hymenopteran venoms, the identification of an enzymatically inactive phospholipase A_2_ (PLA_2_) from the venom of the social wasp *Polybia occidentalis*.

**Methods:**

*P. occidentalis* venom was fractioned by molecular exclusion and reverse phase chromatography. For the biochemical characterization of the protein, 1D and 2D SDS-PAGE were performed, along with phospholipase activity assays on synthetic substrates, MALDI-TOF mass spectrometry and sequencing by Edman degradation.

**Results:**

The protein, called PocTX, was isolated using two chromatographic steps. Based on the phospholipase activity assay, electrophoresis and mass spectrometry, the protein presented a high degree of purity, with a mass of 13,896.47 Da and a basic pI. After sequencing by the Edman degradation method, it was found that the protein showed a high identity with snake venom PLA_2_ homologues.

**Conclusion:**

This is the first report of an enzymatically inactive PLA_2_ isolated from wasp venom, similar to snake PLA_2_ homologues.

## Background

The phospholipases commonly found in wasp venoms are PLA_1_, PLA_2_ and PLB, which are involved in diverse adverse effects during envenoming [1–3]. Phospholipases A_2_ (PLA_2_s) are abundant in the pancreatic juice of mammals and in snake and insect venoms [4]. In bees, this enzyme is the main allergen of the venom, constituting 10-12% of their dry weight [5, 6]. However, this situation is not true for wasp venoms that can present 0.1-1% protein [7, 8]. Few PLA_2_s have been isolated and characterized from wasps, being restricted to incomplete sequences and phospholipase activity on synthetic substrates [9].

These enzymes hydrolyze membrane phospholipids, releasing fatty acids and lysophospholipids as products of the reaction, resulting in the production of lipid mediators, tissue damage and cell death [10, 11]. Disruption of biological membranes by these proteins depends on highly conserved areas among secreted PLA_2_s, such as the Ca^2+^-binding loop, the distribution of disulfide bridges and the presence of a histidine residue at position 48 [10]. However, Lys49 PLA_2_s or homologues from Viperidae snake venoms can disrupt cell membranes and cause myonecrosis through mechanisms that are independent of their catalytic activity [12, 13]. The identification of isoforms of this protein in other organisms, not belonging to group IIA of secreted snake PLA_2_s, shows new gaps regarding the evolutionary process of Lys49 PLA_2_ homologues.

The social wasp *Polybia occidentalis* is endemic in neotropical regions, and is find in almost all Brazilian states [14, 15]. However, few studies have reported the isolation of its molecules. In this study we describe, for the first time, the isolation and characterization of an enzymatically inactive PLA_2_ from *Polybia occidentalis* venom, called PocTX, with high identity with snake venom PLA_2_ homologues.

## Methods

### Materials

The venom of the social wasp *Polybia occidentalis* was kindly provided by Dr. Marta Chagas Monteiro from the Institute of Health Sciences, Federal University of Pará (UFPA). The ethical aspects related to this project were appropriately approved by the Ethics Committee on Animal Use (protocol no. 2012/1), the Ethics Committee of FCFRP-USP (protocol no. 102/2009) and received the Certificate of Presentation for Ethical Appreciation (CAAE: 14204413.5.0000.0011).

### Isolation and biochemical characterization

The crude venom of *P. occidentalis* (100 mg) was solubilized in 50 mM ammonium bicarbonate buffer, pH 8.0, and subjected to size exclusion chromatography in a Sephacryl S200 FF column (1 cm × 40 cm) attached to a GE Akta Purifier HPLC system in an isocratic gradient. The eluted fractions were frozen, lyophilized and tested for phospholipase activity. The fractions of interest were subjected to reverse phase chromatography using a C18 column (25 cm × 4.6 mm, 5 μm, Supelco Discovery) pre-equilibrated with a solution of 0.1% trifluoroacetic acid (TFA) (eluent A) and a linear gradient from 0 to 70% of 99.9% acetonitrile (ACN) and 0.1% TFA (eluent B).

Protein purity was assessed by 1D and 2D polyacrylamide gel electrophoresis with sodium dodecyl sulfate (SDS-PAGE) [16, 17]. Protein quantitation was based on the Bradford method (BioRad) using bovine serum albumin (BSA) as a standard. The gel employed to determine the relative mass of proteins by 1D SDS-PAGE used a discontinuous format at 12.5% under denaturation and reducing conditions. Samples were preheated at 100 °C for 3 min and applied to the wells along with the molecular weight standard (7-175 kDa, BioLabs P7709S). In the electrophoretic run, a current of 15 mA per gel was set along with free voltage for 1 h and 20 min. The gel was stained with Coomassie Blue G-250 and scanned in a GE Image Scanner III.

The 2D electrophoresis consisted of two steps: isoelectric focusing and 1D SDS-PAGE. For the first dimension, the sample was prepared in a rehydration solution (8 M urea, 2% CHAPS, 0.5/2% IPG buffer, 0.002% bromophenol blue and 1 M DTT); this same solution was then incubated with a 7-cm strip (pH 3-10, non-linear) for 12-20 h. After rehydration, the strip was applied to an Ettan IPGphor 3 (GE Healthcare) isoelectric focusing system and later stored at − 80 °C. For the second dimension, the strip was washed with DTT and iodoacetamide diluted in 5 mL of equilibration buffer solution (6 M urea, 2% SDS, 30% glycerol, 50 mM Tris-HCl, pH 7.4, 0.002% bromophenol blue), each. Then, the strip was applied to a 15% polyacrylamide gel. The gel was stained with Coomassie Blue G-250 and scanned in a GE Image Scanner III.

### Phospholipase activity on 4N3OBA

The procedure was performed according to Petrovic et al. [18] with modifications. The phospholipase activity was determined using a solution of 4-nitro-3-octanoyloxy-benzoic acid (4N3OBA) (Enzo Life Sciences, USA) as substrate diluted in 10 mM Tris-HCl buffer pH 8.0, 10 mM CaCl_2_ and 100 mM NaCl and kept refrigerated until it was used. For the activity assay, 190 μL of the reagent 4N3OBA was combined with 10 μL of sample (1 mg/mL) (venom and/or fractions), and immediately incubated in a microplate spectrophotometer (Biotek Eon) at 37 °C. The absorbance was measured at 425 nm for 30 min with kinetic intervals of 1 min. Distilled water and *Bothrops jararacussu* venom were used as controls. The results were submitted to variance analysis followed by Dunnett’s posttest with *p* < 0.05. *Bothrops jararacussu* snake venom was obtained from the serpentarium BioAgents (Batatais, SP, Brazil).

### Obtaining the molecular mass by mass spectrometry

In order to obtain protein molecular masses, a matrix-assisted laser desorption/ionization mass spectrometer (MALDI) with two TOF analyzers (AXIMA TOF-TOF Shimadzu) was used operating in linear mode using sinapinic acid as the ionization matrix. Insulin (5734.5 Da), cytochrome C (12,361.9 Da), apomyoglobin (16,952.2 Da), aldolase (39,212.2 Da) and albumin (66,430.0 Da) were used as calibrants.

### N-terminal sequencing using Edman degradation

N-terminal sequencing of the isolated protein was performed using the Edman degradation technique. The sequence was determined by aPPSQ-33A automated sequencer (Shimadzu, Japan) and later subjected to a similarity search using BLAST software, with subsequent multiple alignment through UniProt.

## Results

**Fig. 1:**
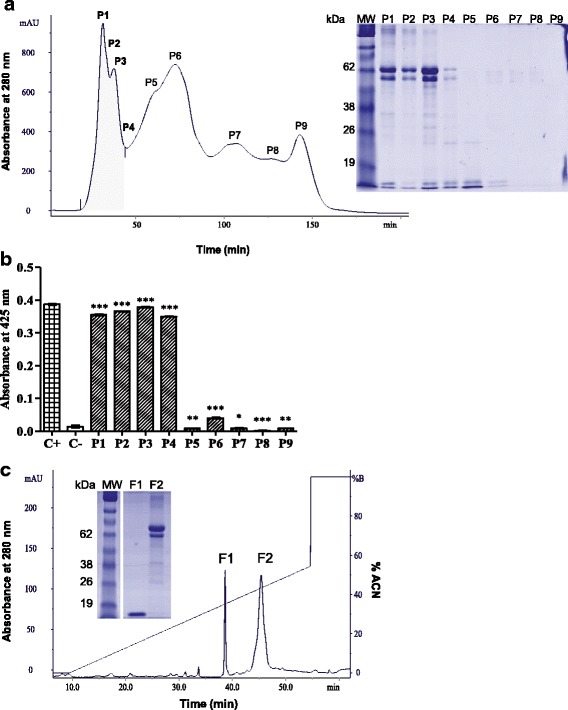
Purification of PocTX. **a** *P. occidentalis* venom (100 mg) was applied to a Sephacryl S200 column, pre-equilibrated with sodium bicarbonate buffer. The eluted fractions were analyzed with 12.5% 1D SDS-PAGE electrophoresis to check the separation profile, where a predominance of relative masses was observed at 65 kDa and 14 kDa. **b** Next, the fractions (10 μg) were tested for their phospholipase activity, among which P1, P2, P3 and P4 had activity on the substrate 4N3OBA. **c** These fractions were mixed and rechromatographed on a reverse phase column, with the elution of two fractions (F1 and F2); upon analysis of the purity of the eluted fractions with 12.5% 1D SDS-PAGE, it was found that one of them showed a single protein band at approximately 14 kDa. The gels were stained with Coomassie Blue G250. The results were expressed as mean ± standard deviation (*n* = 3) and submitted to variance analysis followed by the Tukey posttest. *Significant values when compared to the control groups (*p* < 0.05). C+: positive control – *Bothrops jararacussu* venom. C-: negative control – distilled water

The venom of *P. occidentalis* was subjected to size exclusion chromatography, eluting nine fractions (P1 to P9). One-dimensional electrophoresis of the fractions revealed a profile of protein bands with high and low molecular masses between 62 kDa and 14 kDa in fractions P1 to P4 (Fig. 1a). After a phospholipase activity assay on a specific substrate, it was found that these same fractions were the only ones that degraded the substrate and presented significant activity compared to the positive control (Fig. 1b). Based on this activity, fractions P1 to P4 were pooled and rechromatographed with the elution of two fractions (F1 and F2).

**Fig. 2:**
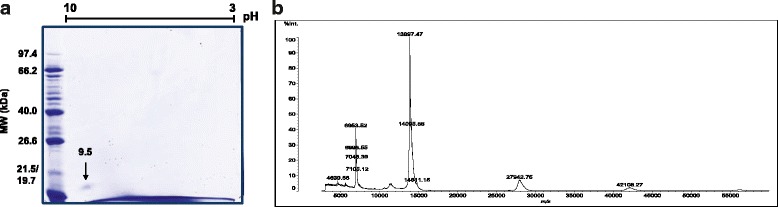
Two-dimensional electrophoresis and mass spectrum of PocTX. **a** PocTX (15 μg) was analyzed by 2D SDS-PAGE electrophoresis using a 7-cm strip, with non-linear pH values from 3 to 10. After isoelectric focusing, the strip was applied to a 15% polyacrylamide gel. The gel was stained with Coomassie Blue G250. **b** Then, PocTX was analyzed by mass spectrometry (MALDI-TOF) to determine its m/z ratio, using sinapinic acid as the ionization matrix, in the mass range of 5000-60,000 m/z

When analyzed with electrophoresis, it was observed that F1 presented a single band of approximately 14 kDa, while F2 contained high molecular weight bands (~ 62 kDa) (Fig. 1c). After an indirect hemolytic activity assay with these fractions through egg yolk emulsion, it was found that the F1 fraction did not present enzymatic activity, while F2 did (data not shown). The observation of a highly pure protein band with the mass of a PLA_2_and no detectable catalytic activity in the tested substrates directed studies to F1. Was this a PLA_2_ homologue? Its purity was confirmed by 2D electrophoresis with the presence of only one spot in the basic region (pI 9.5) (Fig. 2a). Determination of the molecular weight of the protein by mass spectrometry (MALDI-TOF MS) showed the following ions: *m/z* 6963.52 (double charge of the protein), *m/z* 13,897.47 (monomeric form), *m/z* 27,942.75 (dimeric form) and *m/z* 42,108.27 (trimeric form) (Fig. 2b).

**Fig. 3:**
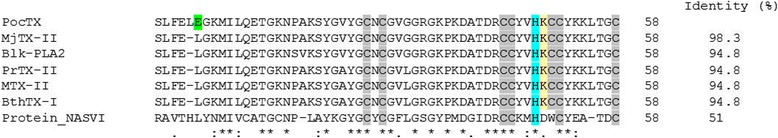
Multiple alignment of the N-terminal sequence of PocTX. After sequencing by Edman degradation (10 μg), the sequence was compared to other snake venom PLA_2_ homologues, including *Bothrops moojeni* (PA2H2_BOTMO, ID: Q91834), *Bothrops leucurus* (PA2HB_BOTLC, ID: P86975), *Bothrops pirajai* (P2H2_BOTPI, ID: P82287), *Bothrops asper* (PA2H2_BOTAS, ID: P24605) and *Bothrops jararacussu* (PA2B1_BOTJR, ID: Q90249), and to an uncharacterized protein from the parasitoid wasp *Nasonia vitripennis* (NASVI_NASVI, ID: K7JAT1). In green, the insertion of a glutamic acid residue in PocTX’s sequence; in blue, a histine residue at position 48; in yellow, a lysine residue at position 49; and in gray, conserved cysteine residues

The Edman degradation method was used to sequence the isolated protein and determine the first 58 amino acid residues from the N-terminal region of the protein. When subjected to similarity and multiple alignment searches, the sequence showed similarity with snake venom phospholipase A_2_ homologues with high identity with the Lys49 PLA_2_ from *Bothrops moojeni* (98.3%), *B. leucurus*, *B. pirajai*, *B. asper* and *B. jararacussu* (94.8%), also presenting significant identity with an uncharacterized protein from the parasitoid wasp *Nasonia vitripennis* (51%) (Fig. 3); the protein was then named PocTX.

## Discussion

For the isolation of PocTX, two chromatographic steps were used: molecular exclusion and reverse phase intercalated with phospholipase activity assays of the collected fractions. After rechromatography of the fractions of interest, a protein with a molecular mass of 13,896.47 Da, devoid of enzymatic activity and with high identity with snake venom Lys49 PLA_2_ homologue, was isolated. Sequence analysis showed a high identity (> 98%) with myotoxin II (MjTX-II) from *B. moojeni*, differing only in the insertion of a glutamic acid residue between residues 5 and 6. Residues conserved in PLA_2_ homologues like Leu5, Gln11, Asn28, Arg34, Lys49, Lys53 and Thr56 are present, along with the cysteine residues in positions 27, 29, 44, 45, 50, 51 and 58 [19].

Some studies have reported the purification of PLA_2_s from wasp venoms, for example: polybitoxins (PbTX I, II, III and IV), glycosylated and highly hemolytic heterodimers with 115-132 kDa [8] from *Polybia paulista* venom; the glycosylated and hemolytic agelotoxin (AgTX), isolated in three states of aggregation – 14, 42 and 74 kDa – from *Agelaia pallipes pallipes* venom [7]; and two PLA_2_s from *P. paulista* venom, with masses of 17,906 and 22,016 Da, one of which shows the presence of glycosylation sites [9].

PocTX is distinct from other PLA_2_s isolated from wasps and Hymenoptera venoms (Asp49 PLA_2_s or enzymatically active) since it showed no identity with proteins isolated and described for this order to date. PLA_2_s found in bees, scorpions, lizards, jellyfish and some human sources are classified within group III of secreted PLA_2_s [10]. These proteins are phylogenetically distinct from groups I and II (which include snake PLA_2_s), but show high similarity in the Ca^2+^-binding loop and the catalytic site region [4, 20], demonstrating that despite having distinct primary sequences, they retain extremely important regions for the implementation of their biological functions.

In a proteomic analysis of the venom of the ant *Solenopsis invicta*, identified several groups of proteins, such as allergens (described for Hymenoptera), PLA_2_s and proteins similar to other animal toxins such as myotoxins, neurotoxins and cytolytic toxins from snakes, arthropods and anemones, respectively [21]. Another study developed by Bouzid et al. [22] demonstrated that the transcriptome of the venom glands of *Tetramorium bicarinatum* presented more than 70% of sequences/transcripts encoded in the list of those not found in databases as well as protein sequences “not belonging to hymenoptera” with similarity to other animal toxins. Similar results were found by Liu et al. [23] who identified in the venom of the killer wasp *Vespa velutina* transcripts/sequences of putative toxins present in snakes and spiders, like C-type lectins and neurotoxins with presynaptic action and activity on ion channels. This demonstrates that many organisms may share components regarded as impassable among phylogenetically distinct species.

PLA_2_s from snakes and hymenopterans are involved in many pathophysiological effects in the event of envenoming. Myotoxic, neurotoxic and hemolytic activities and hemostatic and kidney disorders have been reported [8, 24–30]. The diversity of functions and the presence of different isoforms and phospholipases in hymenopteran venoms show that these proteins are essential for the maintenance of vital functions of these insects. This information is corroborated by Torres et al. [31], who, by means of the giant ant *Dinoponera quadriceps*’s transcriptome, identified the expression of various isoforms of PLA_1_, PLA_2_, PLD and PLB in their venom glands. PLD has not yet been described for hymenopteran venoms.

The isolation of a protein in wasp venoms with similarity with snake venom toxins shows how many gaps remain to be filled in the evolution of animal toxins. There is a variety of underexplored natural sources, proportional to the variety of molecules not yet isolated. PocTX not only allows for new questions about the evolutionary processes that bring such different organisms together, but also leads to inquiries about its involvement in Hymenoptera envenoming.

## Conclusion

This study describes the isolation and physicochemical characterization of PocTX, the first enzymatically inactive PLA_2_ from wasp venom. The protein was purified by two chromatographic steps, intercalated by SDS-PAGE and enzymatic activity. PocTX presented high identity with snake venom Lys49 PLA_2_ homologues, representing a good strategy in understanding the mechanism of action of PLA_2_ homologues in such different venoms.

## Abbreviations

ACN: Acetonitrile
AgTX: Agelotoxin
BSA: Bovine serum albumin
MALDI: Matrix-assisted laser desorption/ionization mass spectrometry
MjTX-II: *B. moojeni* myotoxin II
PbTX: Polybitoxins
PLA_2_: Phospholipases A_2_
PocTX: *Polybia occidentalis* venom PLA_2_
SDS: Sodium dodecyl sulfate
TFA: Trifluoroacetic acid

## References

[1] King TP, Kochoumian L, Joslyn A (1984). Wasp venom proteins: phospholipase A_1_ and B. Arch Biochem Biophys.

[2] Monteiro MC, Romão PRT, Soares AM (2009). Pharmacological perspectives of wasp venom. Protein Pept Lett.

[3] dos Santos LD, Santos KS, Pinto JRA, Dias NB, de Souza BM, dos Santos MF (2010). Profiling the proteome of the venom from the social wasp *Polybia paulista*: a clue to understand the envenoming mechanism. J Proteome Res.

[4] Arni RK, Ward RJ (1996). Phospholipase A_2_--a structural review. Toxicon.

[5] Argiolas A, Pisano JJ (1983). Facilitation of phospholipase A_2_ activity by mastoparans, a new class of mast cell degranulating peptides from wasp venom. J Biol Chem.

[6] Vernon LP, Bell JD (1992). Membrane structure, toxins and phospholipase A_2_ activity. Pharmacol Ther.

[7] Costa H, Palma MS (2000). Agelotoxin: a phospholipase A_2_ from the venom of the neotropical social wasp cassununga (*Agelaia pallipes pallipes*) (hymenoptera-Vespidae). Toxicon.

[8] de Oliveira MR, Palma MS (1998). Polybitoxins: a group of phospholipases A_2_ from the venom of the neotropical social wasp paulistinha (*Polybia paulista*). Toxicon.

[9] dos Santos LD, da Silva Menegasso AR, dos Santos Pinto JRA, Santos KS, Castro FM, Kalil JE (2011). Proteomic characterization of the multiple forms of the PLAs from the venom of the social wasp *Polybia paulista*. Proteomics.

[10] Dennis EA, Cao J, Hsu YH, Magrioti V, Kokotos G (2011). Phospholipase A_2_ enzymes: physical structure, biological function, disease implication, chemical inhibition, and therapeutic intervention. Chem Rev.

[11] Dotimas EM, Hider RC (1987). Honeybee venom. Bee World.

[12] Lomonte B, Rangel J (2012). Snake venom Lys49 myotoxins: from phospholipases A_2_ to non-enzymatic membrane disruptors. Toxicon.

[13] Andrião-Escarso SH, Soares AM, Rodrigues VM, Angulo Y, Díaz C, Lomonte B (2000). Myotoxic phospholipases A_2_ in *Bothrops* snake venoms: effect of chemical modifications on the enzymatic and pharmacological properties of bothropstoxins from *Bothrops jararacussu*. Biochimie.

[14] Mortari MR, Cunha AOS, Carolino ROG, Coutinho-Netto J, Tomaz JC, Lopes NP (2007). Inhibition of acute nociceptive responses in rats after i.c.v. injection of Thr6-bradykinin, isolated from the venom of the social wasp, *Polybia occidentalis*. Br J Pharmacol.

[15] Santos LD, Pieroni M, Menegasso ARS, Pinto JRAS, Palma MS (2011). A new scenario of bioprospecting of hymenoptera venoms through proteomic approach. J Venom Anim Toxins incl Trop Dis.

[16] Laemmli UK (1970). Cleavage of structural proteins during the assembly of the head of bacteriophage T4. Nature.

[17] O’Farrell PZ, Goodman HM, O’Farrell PH (1977). High resolution two-dimensional electrophoresis of basic as well as acidic proteins. Cell.

[18] Petrovic N, Grove C, Langton PE, Misso NL, Thompson PJ (2001). A simple assay for a human serum phospholipase A_2_ that is associated with high-density lipoproteins. J Lipid Res.

[19] Angulo Y, Olamendi-Portugal T, Alape-Girón A, Possani LD, Lomonte B (2002). Structural characterization and phylogenetic relationships of myotoxin II from *Atropoides* (*Bothrops*) nummifer snake venom, a Lys49 phospholipase A2 homologue. Int J Biochem Cell Biol.

[20] Valdez-Cruz NA, Segovia L, Corona M, Possani LD (2007). Sequence analysis and phylogenetic relationship of genes encoding heterodimeric phospholipases A_2_ from the venom of the scorpion *Anuroctonus phaiodactylus*. Gene.

[21] dos Santos Pinto JRA, Fox EGP, Saidemberg DM, dos Santos L, da Silva Menegasso AR, Costa-Manso E (2012). Proteomic view of the venom from the fire ant *Solenopsis invicta* Buren. J Proteome Res.

[22] Bouzid W, Verdenaud M, Klopp C, Ducancel F, Noirot C, Vétillard A (2014). De novo sequencing and transcriptome analysis for *Tetramorium bicarinatum*: a comprehensive venom gland transcriptome analysis from an ant species. BMC Genomics.

[23] Liu Z, Chen S, Zhou Y, Xie C, Zhu B, Zhu H (2015). Deciphering the venomic transcriptome of killer-wasp *Vespa velutina*. Sci Rep.

[24] Lomonte B, Gutiérrez JM (2011). Phospholipases A_2_ from viperidae snake venoms: how do they induce skeletal muscle damage?. Acta Chim Slov.

[25] Angulo Y, Chaves E, Alape A, Rucavado A, Gutiérrez JM, Lomonte B (1997). Isolation and characterization of a myotoxic phospholipase A_2_ from the venom of the arboreal snake *Bothriechis* (*Bothrops*) *schlegelii* from Costa Rica. Arch Biochem Biophys.

[26] de Carvalho ND, Garcia RC, Ferreira AK, Batista DR, Cassola AC, Maria D (2014). Neurotoxicity of coral snake phospholipases A_2_ in cultured rat hippocampal neurons. Brain Res.

[27] Nicolas JP, Lin Y, Lambeau G, Ghomashchi F, Lazdunski M, Gelb MH (1997). Localization of structural elements of bee venom phospholipase A_2_ involved in N-type receptor binding and neurotoxicity. J Biol Chem.

[28] Serrano SM, Reichl AP, Mentele R, Auerswald EA, Santoro ML, Sampaio CA (1999). A novel phospholipase A_2_, BJ-PLA_2_, from the venom of the snake *Bothrops jararaca*: purification, primary structure analysis, and its characterization as a platelet-aggregation-inhibiting factor. Arch Biochem Biophys.

[29] Deshpande PR, Farooq AKK, Bairy M, Prabhu RA (2013). Acute renal failure and/or rhabdomyolysis due to multiple bee stings: a retrospective study. N Am J Med Sci.

[30] Barbosa PSF, Martins AMC, Havt A, Toyama DO, Evangelista JSAM, Ferreira DPP (2005). Renal and antibacterial effects induced by myotoxin I and II isolated from *Bothrops jararacussu* venom. Toxicon.

[31] Torres AFC, Huang C, Chong CM, Leung SW, Prieto-da-Silva ARB, Havt A (2014). Transcriptome analysis in venom gland of the predatory giant ant *Dinoponera quadriceps*: insights into the polypeptide toxin arsenal of hymenopterans. PLoS One.

